# Dynamic evaluation of the cervical spine by kinematic MRI in patients with cervical spinal cord injury without fracture and dislocation

**DOI:** 10.1186/s13018-023-03745-1

**Published:** 2023-03-27

**Authors:** Ao Liu, Nan-Hai Qiu, Xue-Ren Zhong, Xiang Fang, Jun-Jian Liao, Zhi-Peng Zhang, Pei-Feng Zheng, Yong-Yu Hu, Kong-He Hu, Ying-Hui Xiong, Lin-Jun Lu, Xin-Hua Xi, Qiang Wu, Yong-Zheng Bao

**Affiliations:** 1 Department of Spine Surgery, Yuebei People’s Hospital, Guangdong Medical University, Shaoguan City, 512026 Guangdong China; 2 Department of Spine Surgery, Shaoguan First People’s Hospital, Shaoguan City, 512026 Guangdong China

**Keywords:** Cervical spinal cord injury without fracture and dislocation, Magnetic resonance imaging, Kinematic posture, Dynamic evaluation, Imaging measurement

## Abstract

**Background:**

The pattern of changes in the cervical spine and the spinal cord and their dynamic characteristics in patients with cervical spinal cord injury without fracture and dislocation remain unclear. This study aimed to evaluate the dynamic changes in the cervical spine and spinal cord from C2/3 to C7/T1 in different positions by using kinematic magnetic resonance imaging in patients with cervical spinal cord injury without fracture and dislocation. This study was approved by the ethics committee of Yuebei People's Hospital.

**Methods:**

Using median sagittal T2-weighted images for 16 patients with cervical spinal cord injury without fracture and dislocation who underwent cervical kinematic MRI, the anterior space available for the cord, spinal cord diameter, posterior space available for the cord from C2/3 to C7/T1, and Muhle’s grade were determined. The spinal canal diameter was calculated by adding the anterior space available for the cord, spinal cord diameter, and posterior space available for the cord.

**Results:**

The anterior space available for the cord, posterior space available for the cord, and spinal canal diameters at C2/3 and C7/T1 were significantly higher than those from C3/4 to C6/7. Muhle’s grades at C2/3 and C7/T1 were significantly lower than those at the other levels. Spinal canal diameter was lower in extension than in the neutral and flexion positions. In the operated segments, significantly lesser space was available for the cord (anterior space available for the cord + posterior space available for the cord), and the spinal cord diameter/spinal canal diameter ratio was higher than those in the C2/3, C7/T1, and non-operated segments.

**Conclusion:**

Kinematic MRI demonstrated dynamic pathoanatomical changes, such as canal stenosis in different positions, in patients with cervical spinal cord injury without fracture and dislocation. The injured segment had a small canal diameter, high Muhle’s grade, low space available for the cord, and high spinal cord diameter/spinal canal diameter ratio.

## Background

Cervical spinal cord injury without fracture and dislocation is a unique type of spinal cord injury without radiographic abnormalities. It is common in older people (especially those over 46 years old) with minor falls as the major mechanism [1, 2]. Cervical spinal cord injury without fracture and dislocation occurred in 8.7% of adults aged over 65 years in the United States during 2001–2010 [3]. In some patients, the diagnosis of cervical spine injury is delayed because of incomplete radiography or difficulty in visualization [4]. However, an abnormal disc bulge and osteophytes, which contribute to canal stenosis, and abnormal stresses and strains to the spinal cord, which can significantly influence morbidity and prognosis, are often observed in degenerated spine specimens [5, 6]. Approximately 90% of the 42 patients with cervical spinal cord injury without fracture and dislocation showed degenerative changes of the cervical spine such as spondylosis (22 patients) or ossification of the posterior longitudinal ligament (16 patients), or a narrow cervical spinal cord canal (39 patients) [7].

Magnetic resonance imaging (MRI) has been found to be superior to conventional radiography and computed tomography (CT) in the evaluation of pre- or paravertebral hemorrhage or edema, anterior or posterior longitudinal ligament injury, traumatic disc herniation, cord edema, and cord compression [8, 9]. MRI should be performed in the acute period following spinal cord injury before or after surgical intervention to improve the prediction of neurologic and functional outcomes [10]. Even delayed MRI is helpful in determining clinical symptom severity, providing useful information about the state of the spinal cord [11].

In most cases, cervical MRI is performed in a static position, because of which it cannot afford visualization of true pathoanatomical changes in different cervical spine positions. Some previous studies found that cervical neutral static MRI could not demonstrate the changes in the longitudinal ligament and intervertebral disc in the cervical spine during flexion–extension motion. Weisskopf [12] found that there is no correlation between MRI and intraoperative findings; static MRI has limited value in diagnosing traumatic discoligamentous instabilities of the lower cervical spine.

In 1986, cervical kinematic MRI (KMRI) was first used by Koschorek [13] to evaluate the changes in the diameters and lengths of the cervical spinal canal and spinal cord. It was found that adverse mechanical tension might occur in the cervical spinal cord during flexion for the cervical spinal canal and spinal cord lengthen 12.0 mm in average compared with the spinal canal lengthen 28.0 mm in average from flexion to extension position. Recently, the KMRI protocol presented by Pratali et al. [14] was proven to be safe and allowed more complete evaluation of changes in the cervical spine than traditional MRI protocols. KMRI can demonstrate higher Muhle’s stages in the extension position than in the flexion position, but these findings were not related to severe symptoms in patients [15]. KMRI also provides valuable information that is not obtained with neutral-position MRI. For example, based on KMRI in different positions, Lao et al. [16] found that cervical disc bulges were increased remarkably in the extension position compared with those in the neutral position. Approximately 16.4% of the patients without or with < 3 mm of disc bulge in the neutral position presented an increase to ≥ 3 mm bulge in extension; 11.6% of the patients who had a 3–5 mm disc bulge in the neutral view bulged ≥ 5 mm in the extension position.

Currently, the application of KMRI is limited in patients with cervical spinal cord injury without fracture and dislocation owing to the concerns regarding secondary spinal cord injury and the need for patients (especially who suffer high-level cervical spinal cord injury) to maintain the same position for long periods to undergo the examination without electrocardiogram monitoring. In a recent study, we have demonstrated that KMRI can be used for patients with cervical spinal cord injury without fracture and dislocation [17]. Patients with the American Spinal Injury Association Impairment Scale (AIS) grade C, D, or B without respiratory myoparalysis were enrolled and supervised by a spinal surgeon to ensure safety. The AIS grade and Japanese Orthopedic Association (JOA) score did not differ substantially before and after KMRI scans, suggesting that KMRI is a safe and feasible technique for diagnosing cervical spinal cord injury without fracture and dislocation [17]. However, the pattern of changes in the cervical spine and the spinal cord change and their characteristics in patients with cervical spinal cord injury without fracture and dislocation remain unclear.

In the present study, KMRI was used to evaluate the changes of the cervical spine and the spinal cord in patients with cervical spinal cord injury without fracture and dislocation. The objectives of this study were as follows: (1) to demonstrate the imaging measurement changes in disc herniation and canal stenosis at the sub-axial cervical spine levels in neutral, flexion, and extension positions for patients with cervical spinal cord injury without fracture and dislocation and (2) to compare the KMRI characteristics at C2/3, C7/T1, the non-operated segments from C3/4 to C6/7, and the operated segments from C3/4 to C6/7.

## Methods

### Patients

This was a single-institution retrospective study of 16 patients with cervical spinal cord injury without fracture and dislocation who were admitted to Yuebei People’s Hospital between February 2015 and July 2019. All patients presented symptoms of nerve damage after trauma. The patients who met the following inclusion criteria were selected for the study: (i) patients diagnosed with cervical spinal cord injury without fracture and dislocation, but not with respiratory myoparalysis; (ii) patients presenting with no cervical tumor and cervical vertebral fracture or dislocation on cervical radiography or CT; (iii) patients with no medical history of cervical spine injury or operation; and (iv) patients willing to undergo a cervical KMRI scan. Patients who did not meet the inclusion criteria or were managed by other surgeon groups were excluded. All patients underwent surgical management by the same group of surgeons.

### MRI examination

**Fig. 1:**
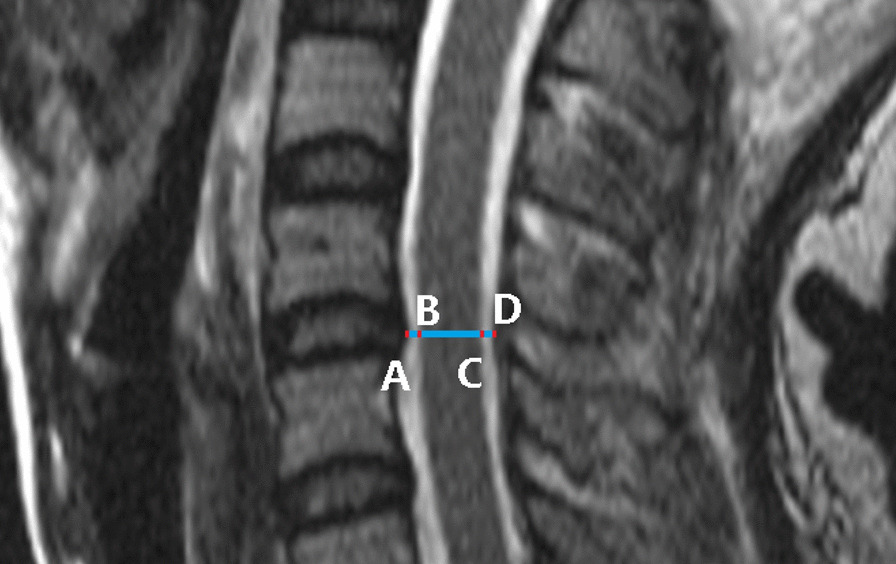
MRI evaluation of spinal cord injury on a T2-weighted median sagittal image acquired from a 30 year-old female patient without fracture and dislocation. Anterior space available for the cord = AB, spinal cord diameter = BC, posterior space available for the cord = CD, spinal canal diameter = AB + BC + CD, and space available for the cord = AB + CD

Static cervical MRI was first performed at the neutral position to confirm the spinal cord injury without fracture and dislocation. Thereafter, KMRI scans were performed in flexion and extension. A 3.0 T scanner (GE Medical Systems, Milwaukee, WI, USA) was used for static and kinematic MRI scans under the supervision of a spinal surgeon. The imaging protocol included T1-weighted and/or T2-weighted sagittal fast spin-echo images obtained by scanning the patient in neutral, flexion, (− 30°), and extension (15°) positions (Fig. 1). The imaging parameters were as follows: (i) repetition time = 860 ms, echo time = 8 ms, thickness = 3.0 mm, and matrix = 216 × 512 for T1-weighted imaging; (ii) repetition time = 2270 ms, echo time = 116 ms, thickness = 3.0 mm, and matrix = 216 × 512 for T2-weighted imaging. The body position was adjusted by placing several rolled towels under the patient’s occipital bone for flexion, or under the back and cervical spine for extension. The flexion or extension angles were decreased if the patient felt any discomfort.

### MRI evaluation

All data obtained from static and kinematic MRI were analyzed using the PACS viewer and imaging system. The anterior space available for the cord, spinal cord diameter, and posterior space available for the cord from C2/3 to C7/T1 were measured on static and kinematic median sagittal T2-weighted images. The spinal canal diameter equals the sum of the anterior space available for the cord, spinal cord diameter, and posterior space available for the cord; the space available for the cord equals the sum of anterior space available for the cord and posterior space available for the cord (Fig. 1). Cervical spinal cord compression from C2/3 to C7/T1 was evaluated using the Muhle’s 4-point grading scale (range, 0–3) (Table 1). All imaging measurements were performed three times by two spinal surgeons independently.

**Table 1:** Muhle’s cervical spinal cord compression grading system [18]

Grade	Cervical spinal canal description
0	Normal width of the spinal canal, with no signs of anterior and posterior subarachnoid space narrowing
1	Partial obliteration of the anterior or posterior subarachnoid space or of both
2	Complete obliteration of the anterior or posterior subarachnoid space or of both
3	Cervical spinal cord compression or displacement or both (pincer effect)

### Surgery procedure

Patients were treated with surgery or conservative therapy based on the static and kinematic imaging results and their own willingness. Anterior cervical discectomy and fusion (ACDF) and posterior lateral mass screw fixation were performed as described in our previous study [17]. For conservative therapy, 125 mL of mannitol was given by intravenous infusion once every 12 h for 3–5 days. After admission, methylprednisolone sodium succinate was administered intravenously with a dose of 500–1000 mg, followed by intravenous infusion of 40 mg every day for 3–5 days. The neurotrophic agent methylcobalamin (1 tablet/time) was given three times a day. The patients were taught to perform limb and joint activity training, and were transferred to the rehabilitation department for rehabilitation therapy according to their willingness.

### Statistical analysis

All obtained data were statistically analyzed using SPSS 24.0 (IBM Corp., Armonk, NY, USA). The data were tested for homogeneity of variance before statistical analysis. A paired *t*-test (homogeneity of variance) and Wilcoxon signed-rank test (heterogeneity of variance) were used for comparisons of the anterior space available for the cord, spinal cord diameter, posterior space available for the cord, and spinal canal diameter among the neutral, flexion, and extension positions. The Mann–Whitney test was used for comparisons of Muhle’s grade between two of the three positions. One-way analysis of variance (homogeneity of variance) and the Mann–Whitney *U* test (heterogeneity of variance) were used for comparing the anterior space available for the cord, spinal cord diameter, posterior space available for the cord, and spinal canal diameter among different sub-axial cervical spine levels in each position. The space available for the cord and the ratio of spinal cord diameter to the spinal canal diameter were compared between the non-operated (C3/4–C6/7) and operated groups using Mann–Whitney *U* test. Two-factor ANOVA was used to determine the effects of cervical disc level and position on the spinal canal diameter. The intra- and interobserver reliability of the MRI measurements were quantified by intraclass correlation coefficient (ICC) values, with a confidence interval of 95%. All significance levels were set at *P* < 0.05.

## Results

### General information of the patients

Sixteen patients (12 males and 4 females) with cervical spinal cord injury without fracture and dislocation from our hospital between February 2015 and July 2019 were included in the study. All the patients underwent KMRI examinations under the supervision of a spinal surgeon. Fourteen patients underwent neutral, flexion, and extension examination; two patients underwent neutral and flexion examination since they could not maintain the position for prolonged durations. The patients were 30–73 years old, with the mean age of 51.1 years old. Clinical symptoms included facial trauma, neck pain, paraplegia, paresthesia, hyperalgesia, sensory loss below the injury level, and dyskinesia. Eleven patients were injured by fall (68.75%), and four were injured in traffic accident (25.00%), while one was injured by heavy pound injury. The JOA score was in the 0–13 range and the AIS grade was B, C, or D.

All patients underwent surgery under management by the same group of surgeons. In total, 12 patients received surgical treatment, while the remaining four patients received conservative therapy. The time from admission to operation ranged from 1 to 3 days, with the mean of 2.25 ± 0.62 days. Seven patients were treated by single-level ACDF, with three at the C3/4 level, one at the C4/5 level, two at the C5/6 level, and one at the C6/7 level. Three patients were treated by double-level ACDF, with two at the C4/5 and C5/6 levels and one at the C5/6 and C6/7 levels. One patient was treated by triple-level ACDF at the C3/4, C5/6, and C6/7 levels. Disc damage and instability of the injured segments were confirmed during operation. Additionally, one patient was treated by posterior C3/4 fusion with lateral mass screws. The operative time ranged from 60 to 130 min (mean: 89.25 ± 22.99 min), and the blood loss ranged from 20 to 100 mL (mean: 42.50 ± 23.69 mL). No surgical complication occurred in this group of patients during the perioperative period.

Furthermore, one patient had no evident cervical spinal cord compression, with minimal signal changes on MRI, and three patients refused to receive surgery. These four patients were given conservative therapy, with no complication during hospitalized period.

### Inter-/intraobserver reliability

For the MRI imaging measurements, interobserver reliability was 0.982 (95% CI 0.980–0.983), while intraobserver reliability was 0.991 (95% CI 0.990–0.992).

### Dynamic evaluation of the cervical spine and the spinal cord

**Fig. 2:**
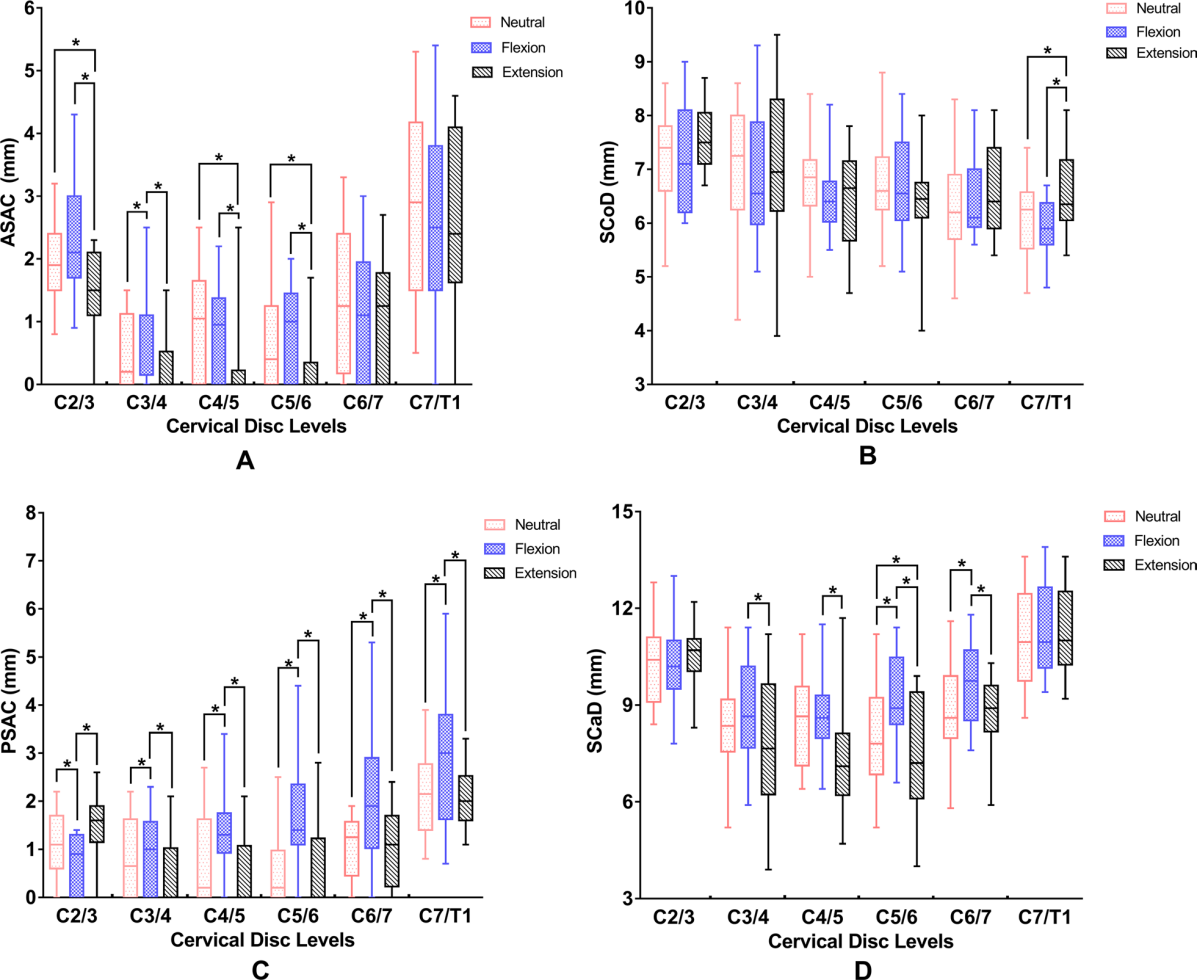
Comparison of **a** anterior space available for the cord (ASAC), **b** spinal cord diameter (SCoD), **c** posterior space available for the cord (PSAC) and **d** spinal canal diameter (SCaD) among the patients in three different positions

Figure 2 shows the changes in the anterior space available for the cord, spinal cord diameter, posterior space available for the cord, and spinal canal diameter at each segment across three different positions. The anterior space available for the cord at C3/4 in the extension position was significantly higher than those in the neutral and flexion positions. In addition, the anterior space available for the cord at C2/3, C4/5, and C5/6 in the extension position were significantly lower than those in the neutral and flexion positions (Fig. 2a). The spinal cord diameter at the C7/T1 level significantly differed between the flexion and extension positions. Meanwhile, the spinal cord diameter at the C7/T1 segment in the extension position was significantly higher than those in the neutral and flexion positions (Fig. 2b). The posterior space available for the cord in the flexion position was significantly lower than those in the other two positions at C2/3, while an inverse trend was observed from C3/4 to C7/T1 levels (Fig. 2c). The spinal canal diameter value at C4/5 in the extension position was significantly lower than those in the neutral and flexion positions, while the spinal canal diameter values at C5/6 and C6/7 in the flexion position were significantly higher than those in the other two positions (Fig. 2d).

**Fig. 3:**
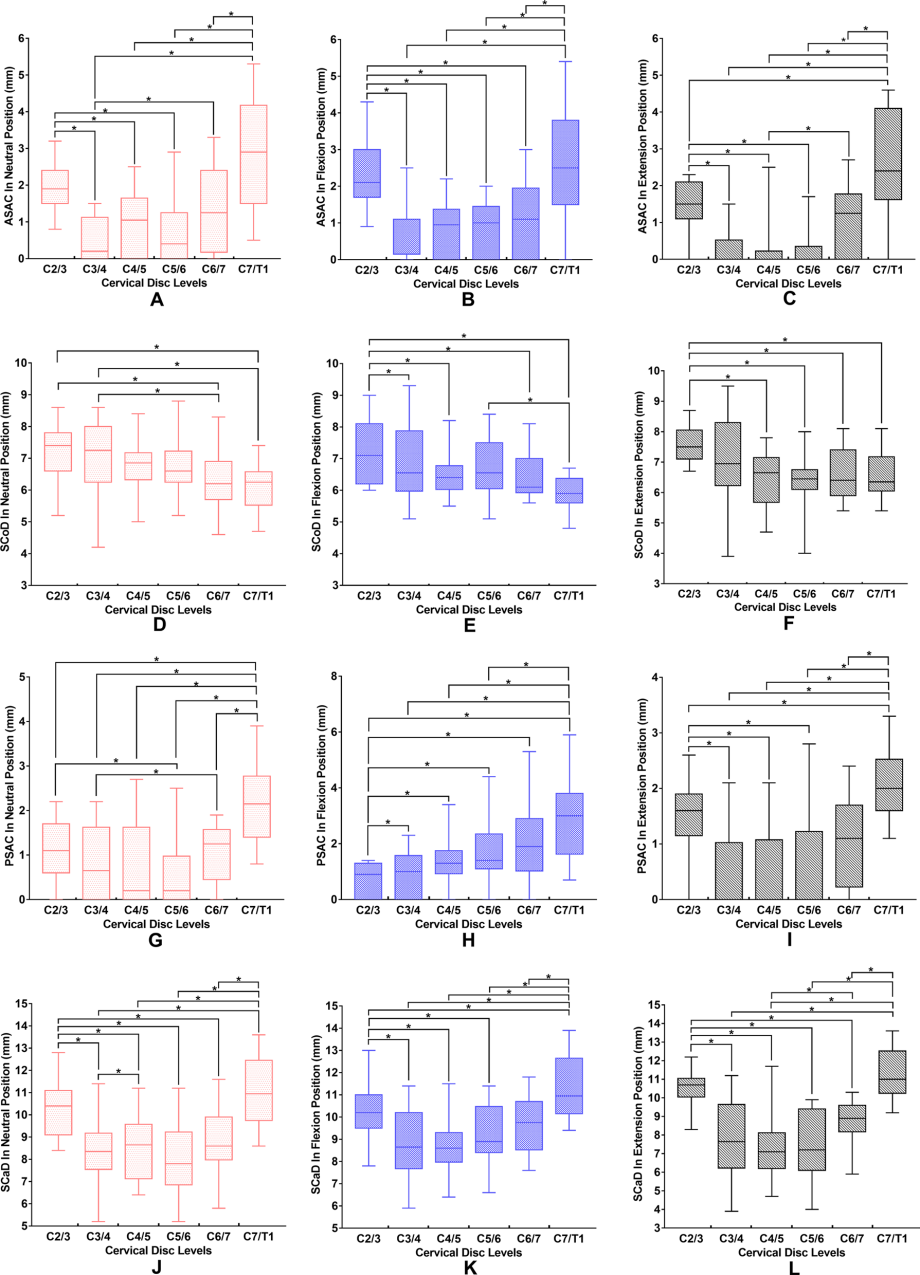
Comparison of **a**–**c** ASAC, **d**–**f** SCoD, **g**–**i** PSAC and **j**–**l** SCaD at different levels in each position

Figure 3 shows the changes in the anterior space available for the cord, spinal cord diameter, posterior space available for the cord, and spinal canal diameter at different levels in each position. The anterior space available for the cord at C2/3 and C7/T1 were much higher than those from C3/4 to C6/7 irrespective of the position (Fig. 3a–c). C2/3 showed the largest spinal cord diameter, and C3/4 showed the second-largest spinal cord diameter in all cases (Fig. 3d–f). In addition, the spinal cord diameter at C5/6 was significantly higher than that at C7/T1 in the flexion position (Fig. 3e). The posterior space available for the cord at C2/3 was significantly lower than those from C3/4 to C7/T1 in the flexion position (Fig. 3h), but higher than those from C3/4 to C5/6 in the extension position (Fig. 3i). Meanwhile, the posterior space available for the cord at C7/T1 was significantly higher than those from C2/3 to C5/6 in all three positions (Fig. 3g–i). The spinal canal diameter values at C2/3 and C7/T1 were higher than those from C2/3 to C6/7 in each position (Fig. 3j–l). The spinal canal diameter values at C4/5 and C5/6 were lower than those at other levels in the extension position (Fig. 3l). Two-factor ANOVA showed that spinal canal diameter was affected by cervical disc level and position. The spinal canal diameter values at C2/3 and C7/T1 were higher than those from C2/3 to C6/7, irrespective of MRI position (*P* < 0.001). The spinal canal diameter values in the flexion position were higher than those in extension, irrespective of the cervical disc level (*P* < 0.001). No interaction was observed between cervical disc level and MRI positions (*P* = 0.27).

**Fig. 4:**
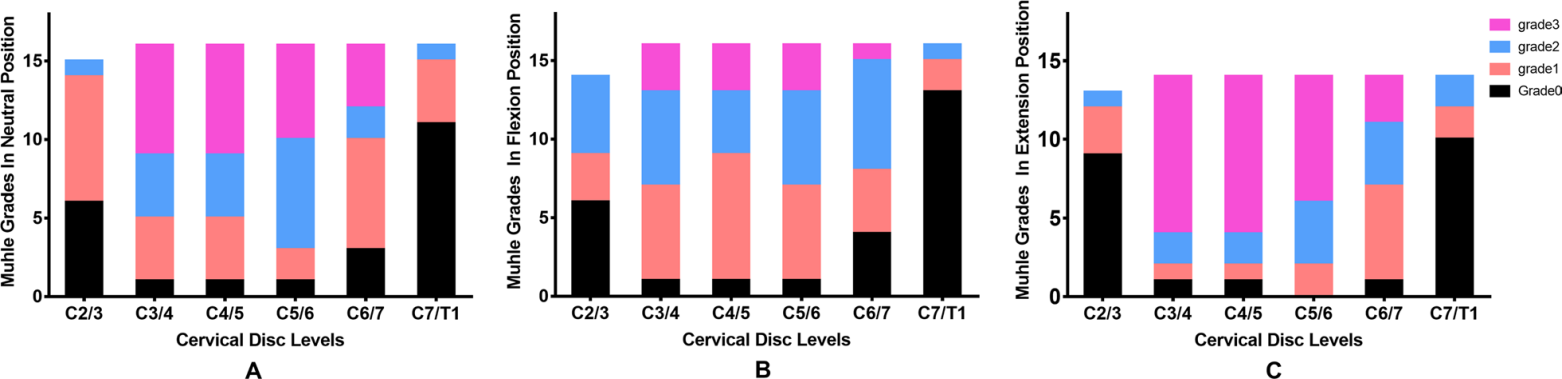
Comparison of Muhle’s grade at different levels in the **a** neutral, **b** flexion and **c** extension positions

Significant differences in Muhle’s grade were observed between the flexion and extension positions at C3/4 and C4/5. In addition, Muhle’s grade at C2/3 in the neutral position was significantly lower than those from C3/4 to C5/6 in the neutral position and from C3/4 to C6/7 in the extension position. Generally, Muhle’s grades at C2/3 and C7/T1 were lower than those from C3/4 to C6/7 in the three different positions (Fig. 4).

**Fig. 5:**
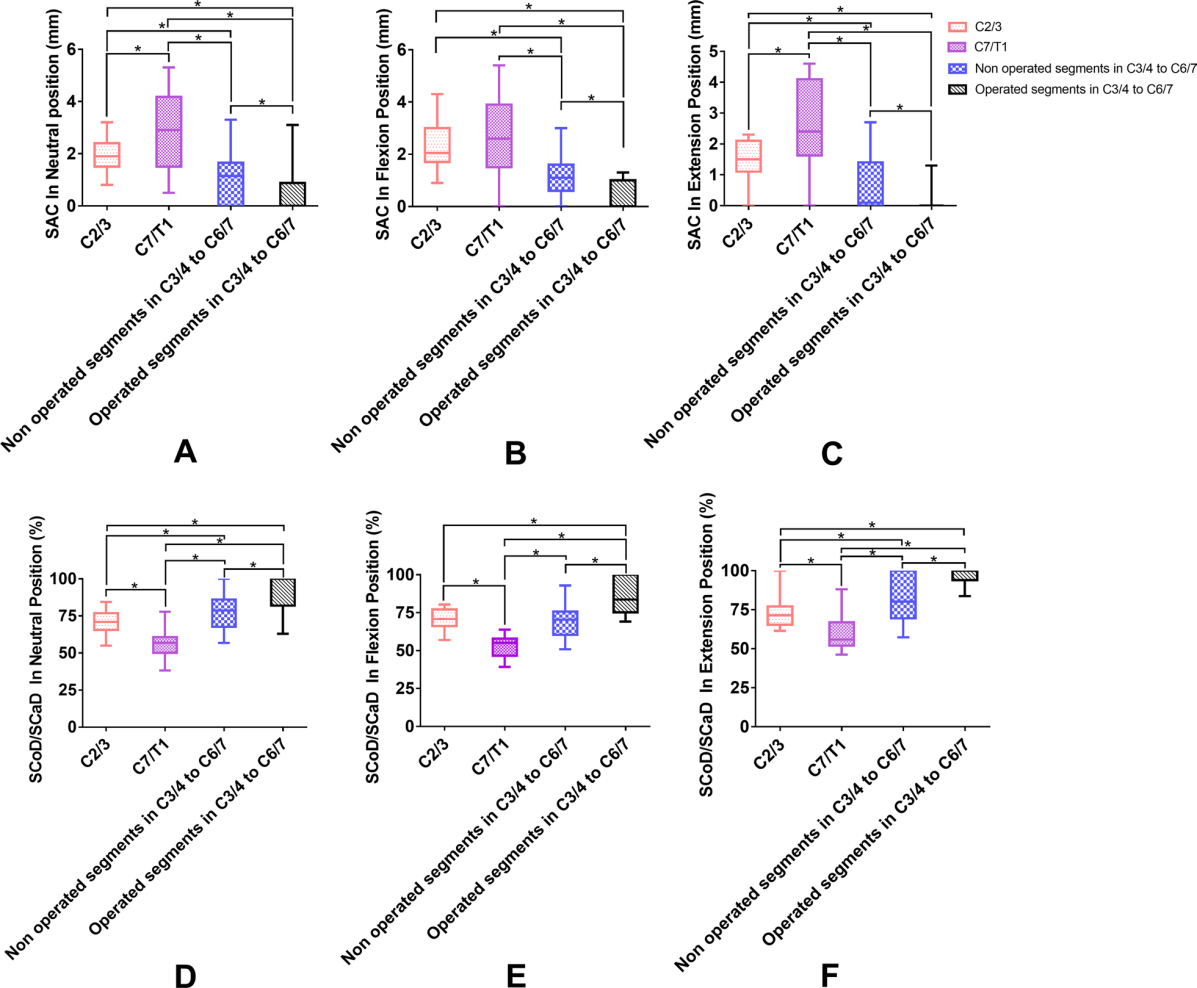
Comparison of **a**–**c** the space available for the cord (SAC) and **d**–**f** the SCoD/SCaD ratio between different groups in the three positions

The segments were divided into C2/3, C7/T1, non-operated segments in C3/4–C6/7, and operated segments in C3/4–C6/7 to further analysis. Figure 5a–c shows that C7/T1 had the largest space available for the cord compared with the other groups, and the space available for the cord in the operated segments was significantly lower than those at C2/3, C7/T1, and the non-operated segments in three different positions. Figure 5d–f shows that the spinal cord diameter/spinal canal diameter ratio in the operated segments was significantly higher than those at C2/3, C7/T1, and the non-operated segments in three different positions.

### Typical case

**Fig. 6:**
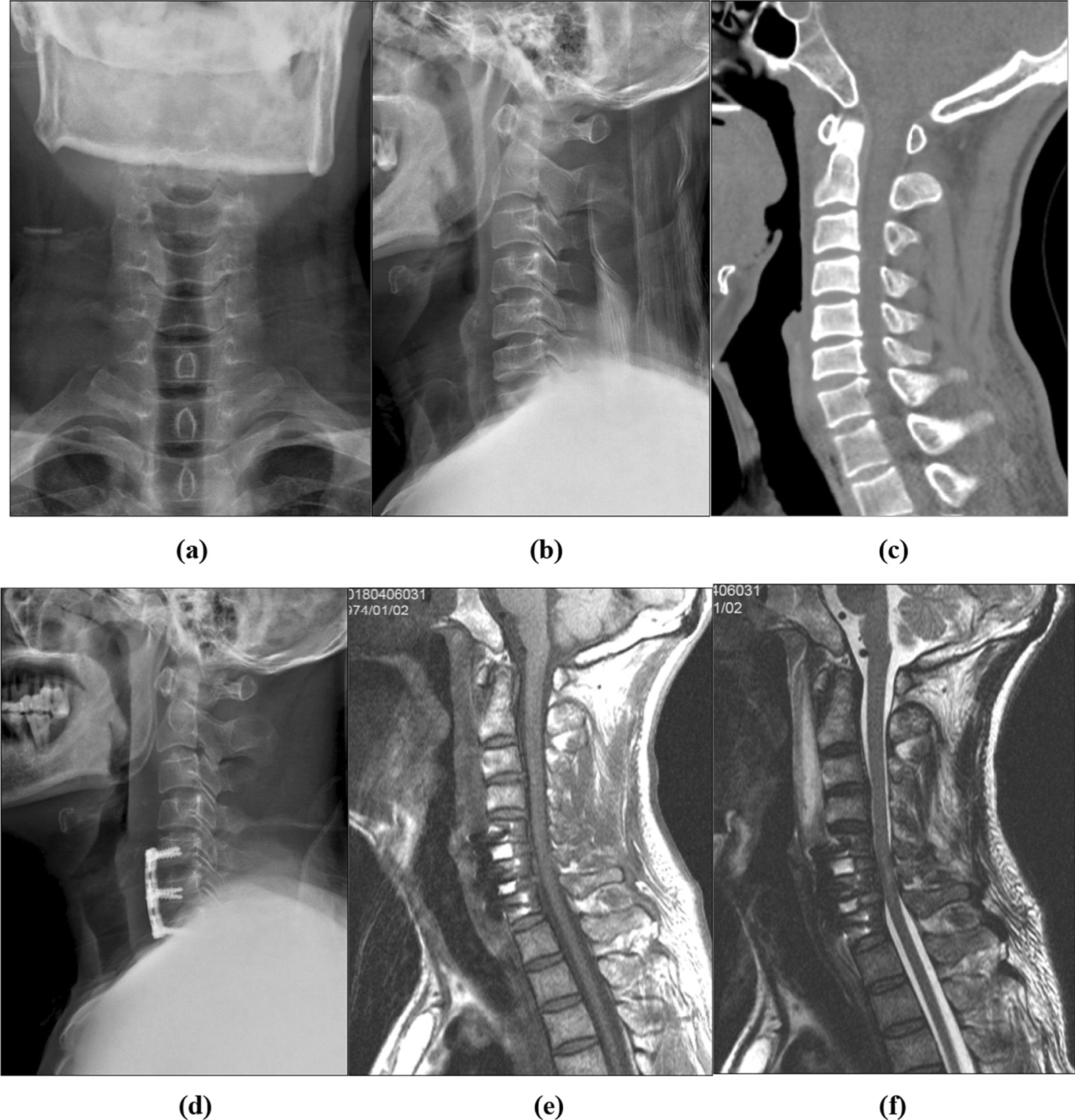
Cervical radiography (**a**, **b**) and CT (**c**) showed reduced cervical physiological curvature and mild hyperostosis with no distinctive fractures in a 45 year-old male patient (case 2). Post-operative reexamination by radiography (**d**) showed the favorable position of internal fixation and no apparent compression of the spinal cord (**e**, **f**)

**Fig. 7:**
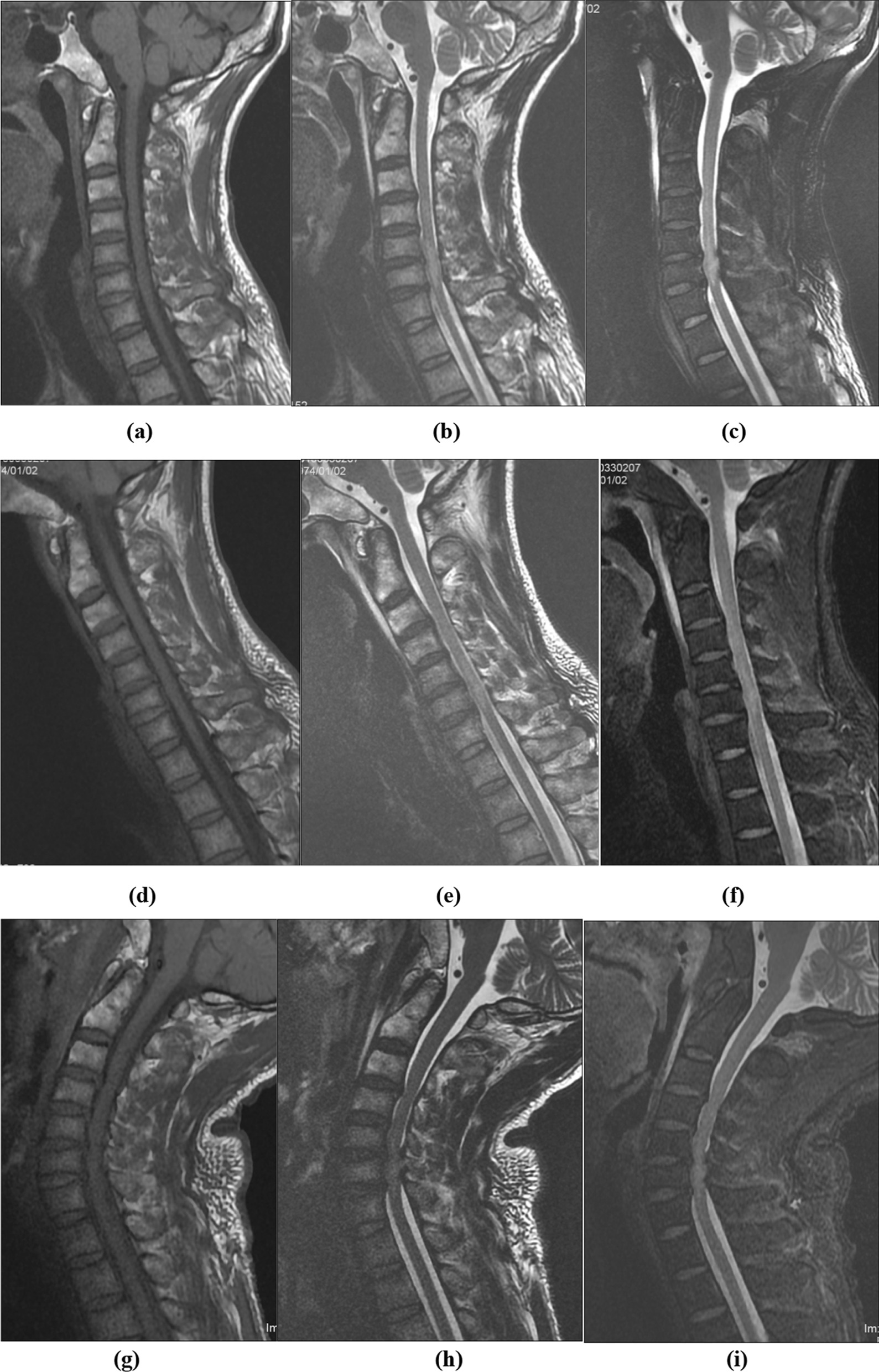
Cervical neutral sagittal T1-weighted MRI (**a**) confirmed the absence of vertebral body fracture and no signal of hemorrhage in the spinal cord of case 2; T2-weighted (**b**) and fat-saturated (**c**) imaging revealed abnormal signals of injury in the spinal cord from the C5 vertebral body to the cervical 6/7 disc level, in addition to disc herniation with spinal cord compression at C5/6 and C6/7. Cervical flexion MRI (**d**–**f**) showed reduced disc herniation without evident compression of the spinal cord at C5/6 and C6/7. Cervical extension MRI (**g**–**i**) showed aggravated disc herniation with spinal cord compression and canal stenosis at C5/6 and C6/7

A 45 year-old male patient was injured due to falling off the bicycle. He complained of neck pain accompanied by limited range of motion of limbs for two days before admission to our hospital. His AIS grade was C and the JOA score was 4 on admission. Cervical radiography and CT scans revealed reduced cervical physiological curvature and mild hyperostosis without apparent fractures (Fig. 6a–c). Cervical neutral MRI confirmed the absence of vertebral body fracture and no signal of hemorrhage in the spinal cord in T1-weighted imaging (Fig. 7a); there were abnormal signals of injury in the spinal cord from the C5 vertebral body to the cervical 6/7 disc level, while disc herniation with spinal cord compression appeared at C5/6 and C6/7 on T2-weighted and fat-saturated images (Fig. 7b, c). Flexion MRI indicated reduced disc herniation without evident spinal cord compression at C5/6 and C6/7 (Fig. 7d–f), whereas extension MRI revealed aggravated disc herniation with spinal cord compression and canal stenosis at C5/6 and C6/7 (Fig. 7g–i). Therefore, the patient was treated by ACDF with iliac bone grafting at C5/6 and C6/7. Post-operative reexamination by radiography showed that the position of internal fixation was favorable (Fig. 6d) and there was no apparent compression of the spinal cord (Fig. 6e, f). The AIS grade was C and the JOA score was 7 one week after operation. Three years later, the AIS grade and JOA score changed to D and 17, respectively.

## Discussion

In this study, we retrospectively analyzed the records of patients with cervical spinal cord injury without fracture and dislocation from a single institution. Falls were the most common cause of spinal cord injuries in our patients with cervical spinal cord injury without fracture and dislocation, consistent with previous studies [2, 3]. Furthermore, according to a kinematical study on cadavers and volunteers, there is a tendency for injury in the upper cervical spine at higher trauma accelerations [19].

The KMRI evaluations showed that the anterior space available for the cord, posterior space available for the cord, and spinal canal diameter at C3/4–C6/7, but not C2/3 or C7/T1, showed a decreasing trend from flexion to extension, whereas the spinal cord diameter appeared to be relatively stable in different positions. The ligamentum flavum shows stretching in flexion, and the disc protrudes posteriorly into the canal, while the ligamentum flavum protrudes anteriorly in extension in comparison with the neutral position [20]. Muhle’s grade assessments also indicated that spinal cord compression was relieved in flexion but aggravated in the extension position among the patients with cervical spinal cord injury without fracture and dislocation. Similar changes were reported by Breig et al. [21], who performed biomechanical research of the cervical spine and showed that cervical canal and cord length change according to the physiological movements of the cervical spine but do not produce any abnormal stresses and strains in the nervous tissue. Xiong et al. [22] found similar changes in symptomatic patients with mild cervical myelopathy spondylosis. These observations indicate that the available space for the spinal cord decreases from flexion to extension in patients with cervical spinal cord injury without fracture and dislocation or cervical myelopathy.

Generally, the spinal cord diameters at the upper levels (C2/3 and C3/4) were markedly higher than those at the lower levels (C6//7 and CT/71). The anterior space available for the cord, posterior space available for the cord, and spinal canal diameter at C2/3 and C7/T1 were substantially higher than those at other levels, especially C4/5 and C5/6. These results are similar to the previous KMRI findings for cervical myelopathy [14, 16, 23]. Chen et al. [24] found that that cervical spine injury may be caused in three distinct periods and the lower cervical vertebrae are injured in hyperextension when the spine forms an S-shaped curve before the neck is fully extended. In the present study, we found that the segments from C3/4 to C6/7 had narrower spinal canal diameters, often with disc herniation or ligamentum flavum hypertrophy contributing to canal stenosis, which probably explains the preponderance of cord injury in the extension position. In all injured discs, the C3/4 and C4/5 levels accounted for 25% (5/20) each, and the C5/6 level made up 35% (7/20), while the C6/7 level makes up 15% (3/20).

A disc-level canal diameter less than 8 mm is considered to be a risk factor for acute cervical spinal cord injury after minor trauma [25]. Chen et al. [26] found that the average disk bulb changed 10.8% of the canal diameter, and the ligamentum flavum bulge changed 24.3% of the canal diameter, resulting from flexion–extension loading. The canal diameter narrowing during whiplash could squeeze the cord between the posterior aspect of the upper vertebral body and the lamina of the lower vertebra, which was described as a “pincer” mechanism [27]. In the present study, almost all spinal canal diameter values in the operated segments from C3/4 to C6/7 were less than 8 mm in three different positions. Moreover, Muhle’s grade is a comprehensive indicator of changes in the available space for the spinal cord caused by canal stenosis, disc herniation, and ligamentum flavum. Here, grade 3 was more common at the C3/4, C4/5, and C5/6 levels in the neutral position, which decreased in the flexion position and increased in the extension position among the patients with cervical spinal cord injury without fracture and dislocation.

In this study, there were no operated segments at the C2/3 or C7/T1 level in the patients with cervical spinal cord injury without fracture and dislocation. The segments were divided into C2/3, C7/T1, non-operated (C3/4–C6/7), and operated groups for further analysis. The operated group included three segments at C3/4, three at C4/5, five at C5/6, and two at C6/7. C4/5 had the lowest spinal canal diameter and C5/6 had the second-lowest spinal canal diameter, both in the neutral and extension positions. C4/5 (3/13) and C5/6 (5/13) had higher risks of injury than the other levels, which has been corroborated by a previous kinematic analysis [28]. Ito et al. [27] used a biofidelic model to simulate whiplash and found that spinal cord injury during whiplash is unlikely to occur in patients with normal average canal diameters. The relative risk for the incidence of traumatic cervical spinal cord injury at the C3/4 segment with cervical spinal canal stenosis was calculated as 124.5:1 in comparison with healthy volunteers [29]. Furthermore, C7/T1 showed the largest space available for the cord, and C2/3 showed the second-largest space available for the cord. The space available for the cord in the non-operated group were also markedly higher than those in the operated group. The larger space available for the cord could protect the cervical spinal cord from injury.

With regard to the spinal cord diameter/spinal canal diameter ratio, a high value is considered a risk factor for the development of cervical spinal cord compression [30]. In our study, the spinal cord diameter/spinal canal diameter ratio increased gradually from the flexion to extension position in all groups. The operated group showed the largest spinal cord diameter/spinal canal diameter ratio in three different positions, with the value in the extension position being significantly higher than those at C2/3 and C7/T1. Significant differences were also observed between the operated and non-operated groups. Accordingly, low space available for the cord and a high spinal cord diameter/spinal canal diameter ratio seem to be risk factors for spinal cord injury in the patients with cervical spinal cord injury without fracture and dislocation. Muhle’s grade in the operated segments was higher than those in the non-operated segments at all levels. High Muhle’s grade may also be a risk factor for cervical spinal cord injury in this patient population, but further research is required to confirm this point.

Our study had some limitations. First, the sample size was small and the study included patients with mild spinal cord injury (mostly AIS grades C and D) that could not represent the kinematic changes in severe cervical spinal cord injury without fracture and dislocation. Clinicians should be cautious to operate on patients with acute injuries with relatively normal MRIs just based on KMRI parameters. Second, this was a single-institution study with a retrospective design. Multi-institution prospective studies are encouraged to verify the results of this study.

## Conclusion

To the best of our knowledge, this is the first imaging evaluation of the cervical spine and the spinal cord via KMRI in patients with cervical spinal cord injury without fracture and dislocation. Based on the results, KMRI can reveal pathoanatomical changes such as canal stenosis in the flexion and extension positions in order to determine the need for and specific surgical intervention. The injured segment is characterized by a small canal diameter, a high Muhle’s grade, a low space available for the cord, and a high ratio of spinal cord diameter to spinal canal diameter.

## Abbreviations

ACDF: Anterior cervical discectomy and fusion
AIS: American Spinal Injury Association Impairment Scale
CT: Computed tomography
MRI: Magnetic resonance imaging
KMRI: Kinematic magnetic resonance imaging
FSE: Fast spin echo
JOA: Japanese Orthopedic Association
TE: Echo time
TR: Repetition time
